# Left Axis Deviation in Brugada Syndrome: Vectorcardiographic Evaluation during Ajmaline Provocation Testing Reveals Additional Depolarization Abnormalities

**DOI:** 10.3390/ijms22020484

**Published:** 2021-01-06

**Authors:** Martijn H. van der Ree, Jeroen Vendrik, Jan A. Kors, Ahmad S. Amin, Arthur A. M. Wilde, Hanno L. Tan, Pieter G. Postema

**Affiliations:** 1 Heart Center, Department of Clinical and Experimental Cardiology, Amsterdam UMC, University of Amsterdam, Cardiovascular Sciences, Meibergdreef 9, 1105 AZ Amsterdam, The Netherlands; mailto:m.h.vanderree@amsterdamumc.nl (M.H.v.d.R.); j.vendrik@amsterdamumc.nl (J.V.); a.s.amin@amsterdamumc.nl (A.S.A.); a.a.wilde@amsterdamumc.nl (A.A.M.W.); h.l.tan@amsterdamumc.nl (H.L.T.); 2 Department of Medical Informatics, Erasmus MC, University Medical Center Rotterdam, Doctor Molewaterplein 40, 3015 GD Rotterdam, The Netherlands; j.kors@erasmusmc.nl; 3 Netherlands Heart Institute, Moreelsepark 1, 3511 EP Utrecht, The Netherlands

**Keywords:** Brugada syndrome, vectorcardiogram, left axis deviation, ventricular arrhythmias

## Abstract

Patients with Brugada syndrome (BrS) can show a leftward deviation of the frontal QRS-axis upon provocation with sodium channel blockers. The cause of this axis change is unclear. In this study, we aimed to determine (1) the prevalence of this left axis deviation and (2) to evaluate its cause, using the insights that could be derived from vectorcardiograms. Hence, from a large cohort of patients who underwent ajmaline provocation testing (*n* = 1430), we selected patients in whom a type-1 BrS-ECG was evoked (*n* = 345). Depolarization and repolarization parameters were analyzed for reconstructed vectorcardiograms and were compared between patients with and without a >30° leftward axis shift. We found (1) that the prevalence of a left axis deviation during provocation testing was 18% and (2) that this left axis deviation was not explained by terminal conduction slowing in the right ventricular outflow tract (4th QRS-loop quartile: +17 ± 14 ms versus +13 ± 15 ms, nonsignificant) but was associated with a more proximal conduction slowing (1st QRS-loop quartile: +12[8;18] ms versus +8[4;12] ms, *p* < 0.001 and 3rd QRS-loop quartile: +12 ± 10 ms versus +5 ± 7 ms, *p* < 0.001). There was no important heterogeneity of the action potential morphology (no difference in the ventricular gradient), but a left axis deviation did result in a discordant repolarization (spatial QRS-T angle: 122[59;147]° versus 44[25;91]°, *p* < 0.001). Thus, although the development of the type-1 BrS-ECG is characterized by a terminal conduction delay in the right ventricle, BrS-patients with a left axis deviation upon sodium channel blocker provocation have an additional proximal conduction slowing, which is associated with a subsequent discordant repolarization. Whether this has implications for risk stratification is still undetermined.

## 1. Introduction

In patients suspected of Brugada syndrome (BrS), documenting the spontaneous type-1 BrS-ECG or, after, provocation testing with a cardiac sodium channel blocker is a required criterion for the BrS-diagnosis [1]. During such provocation testing, the QRS axis may deviate towards the left [2,3]. As the right ventricular outflow tract (RVOT) is a critical area in the development of the type-1 BrS-ECG and its associated malignant arrhythmias [1,4], this left axis deviation may be caused, among others, by exaggerated conduction slowing in the RVOT exceeding the conduction slowing that is already associated with the development of the type-1 ECG [1,4,5]. This could result in a diminished rightward vector and consequently a more pronounced and leftward vector, resulting in a leftward axis deviation [1]. Alternatively, this leftward deviation may be due to more proximal conduction abnormalities. Currently, the prevalence of a left axis deviation in drug-induced BrS and its origin are unknown. Importantly, when the underlying pathophysiological mechanisms underlying the ECG variations in BrS are unraveled, this could contribute to risk stratification.

While the 12-lead ECG is unsuitable for determining the origin of a leftward axis deviation, vectorcardiography provides more three-dimensional electrophysiological data and consequently more spatiotemporal information [4,6,7]. For this reason, the vectorcardiogram is potentially able to distinguish between conduction slowing in the RVOT and more proximal conduction abnormalities, thereby providing the opportunity to discover the origin of the left axis deviation. Furthermore, the vectorcardiogram is also able to provide additional information on repolarization characteristics [4,8,9], which could provide additional insights on the electrophysiological effects of axis deviations.

In this study, we evaluated vectorcardiograms of a large cohort of patients suspected of BrS who underwent provocation testing in order to (1) determine the prevalence of a left axis deviation in patients with a positive ajmaline test result and (2) to evaluate the cause of this left axis deviation.

## 2. Results

### 2.1. Baseline Characteristics

**Figure 1:**
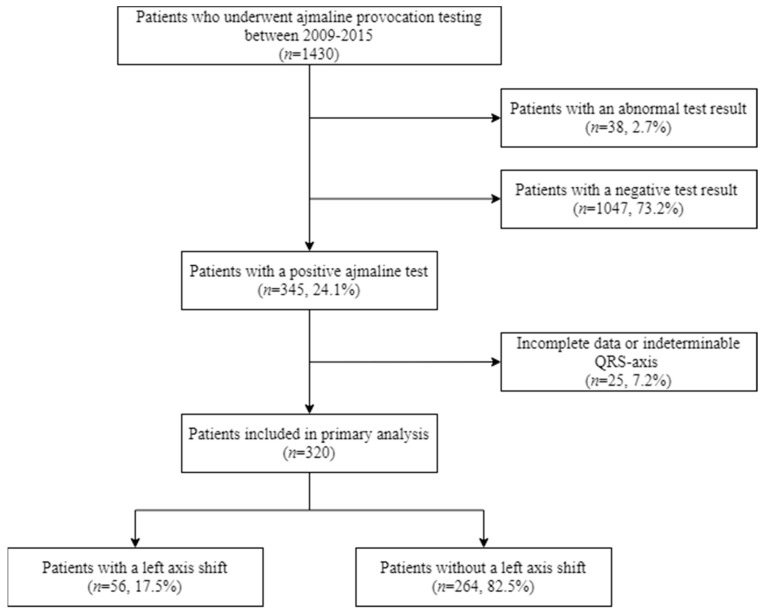
Flowchart of the patient selection.

Figure 1 shows the flowchart of the selection of patients. In total, 345 patients in whom ajmaline testing elicited a type-1 BrS-ECG (positive test) were identified from a total cohort of 1430 patients who underwent provocation testing (24.1%). In total, 320 of these 345 (92.8%) patients were included for this study, and 25 (7.2%) patients were excluded due to incomplete data or an indeterminable axis. The prevalence of a left axis deviation in patients with a positive test and determinable QRS-axis was 17.5% (*n* = 56/320). Patients with a left axis deviation at baseline were more often female and were significantly shorter and lighter; the BMI, however, did not significantly differ (Table 1). The age, histories of (possible) arrhythmias, the indication for testing and the administered ajmaline dose, as well as the percentage of the ajmaline maximal target dose, did not differ (Table 1). The presence of an *SCN5A* mutation was also not significantly different between the groups. Please note that genetic testing was not performed in all patients, in particular when genetic testing in a family member had already revealed the absence of a potentially causative mutation. The baseline ECG parameters are presented in Table 1.

### 2.2. Baseline Vectorcardiogram Parameters

Patients with a positive test and a left axis deviation upon provocation testing had, at baseline, shorter QRS-durations when compared to patients without a left axis deviation upon provocation testing (100 ± 14 ms versus 105 ± 15 ms, *p* < 0.05). In the 3rd quartile of the QRS-loop, conduction was slightly faster in patients with a left axis deviation upon provocation (10[8;12] ms versus 12[10;14] ms, *p* < 0.05). There was no difference in the depolarization–repolarization interaction in both groups (Table 2, QRS-T angle and ventricular gradient).

### 2.3. Vectorcardiogram Parameters at Peak Ajmaline Dose

#### 2.3.1. Depolarization Abnormalities

**Figure 2:**
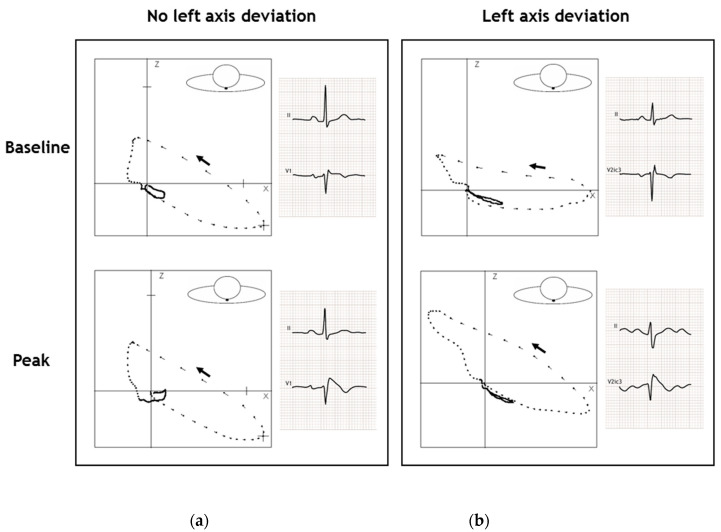
The QRS-T loop in the transverse plane of two representative patients at baseline and ajmaline peak. (**a**) Patient without a left axis deviation. The QRS-axis in the frontal plane changed 3° to the left. At baseline, the QRS-duration was 94 ms and increased to 106 ms at ajmaline peak; the changes in the duration of the quartiles of the QRS-loops were: q1 +0 ms, q2 +2 ms, q3 +2 ms and q4 +8 ms. The QRS-T angle changed with –19° from 27° at baseline to 8° at ajmaline peak; (**b**) Patient with a left axis deviation. The QRS-axis in the frontal plane changed 60° to the left. At baseline, the QRS-duration was 92 ms and increased to 138 ms at ajmaline peak; the changes in the duration of the quartiles of the QRS-loops were: q1 +14 ms, q2 -2 ms, q3 +18 ms and q4 +16 ms. The QRS-T angle changed with +60° from 40° at baseline to 100° at ajmaline peak. Dashes of the QRS-T loop: 2-ms intervals. The black arrow indicates the QRS-loop direction.

**Figure 3:**
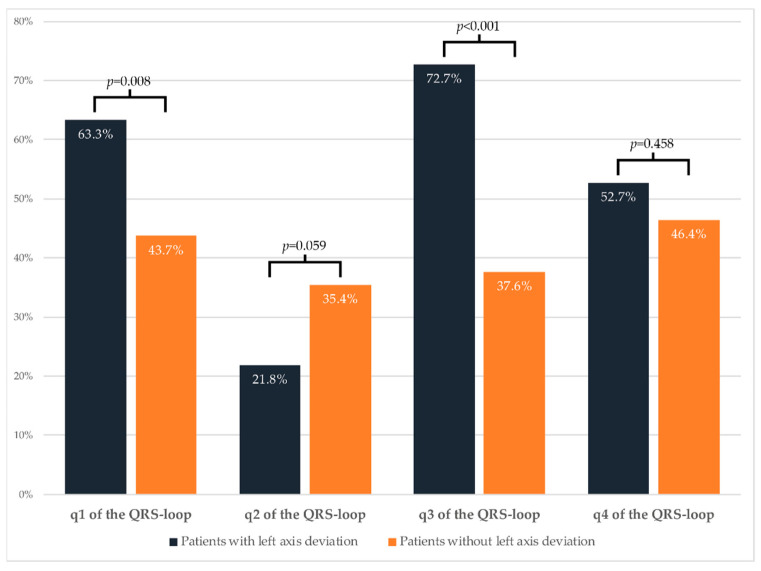
Proportion of patients with conduction slowing above the median value of the entire cohort.

At the peak ajmaline dose, progressive conduction slowing had occurred in all four quartiles in both groups (Table 2). Patients with a left axis deviation showed more of a conduction delay when compared to patients without a left axis deviation (QRS duration: +43 ± 17 ms versus +30 ± 21 ms, *p* < 0.001). This conduction delay in patients with a left axis deviation occurred primarily in the first (+12[8;18] ms versus +8[4;12] ms, *p <* 0.001) and third (+12 ± 10 ms versus + 5 ± 7 ms, *p <* 0.001) quartiles. In the second quartile, conduction slowing was less pronounced in patients with a left axis deviation (+2[−2;6] ms versus +6[2;8] ms, *p* <0.001). Noticeably, the conduction delay in the fourth quartile was similar in both groups (Table 2). In addition, there were no differences in the amount of maximal right-precordial ST-elevation (measured at the J-point) between the two groups. In Figure 2, the QRS-T loops of two representative patients at baseline and ajmaline peak are shown; from this figure, the conduction slowing at the peak ajmaline dose when compared to baseline (especially in the patient with left axis deviation) can be appreciated. Figure 3 shows the proportion of patients with a left axis deviation and an increase of the conduction intervals above the median value of the cohort. For the first (63.3% versus 43.7%, *p <* 0.05) and third quartiles (72.7% versus 37.6%, *p <* 0.001) this proportion was significantly higher compared to patients without a left axis deviation (Figure 3). For the second quartile, in contrast, the proportion of patients with conduction slowing was larger in the patients without a left axis deviation (21.8% versus 35.4%, *p* = 0.059). The proportion of patients with conduction slowing above the median of the entire cohort in the fourth quartile was similar in both groups (52.7% versus 46.4%, *p* = 0.458) (Figure 3). 

#### 2.3.2. Repolarization Abnormalities

The spatial QRS-T angle significantly differed at the peak ajmaline dose (Table 2): in patients with a left axis deviation, the spatial QRS-T angle rose to borderline abnormal values (>105–135°) whilst remaining normal in the patients without a left axis deviation (122[59;147]° versus 44[25;91]°, *p <* 0.001). The heterogeneity of the action potential morphology, as expressed by the ventricular gradient, did not significantly change or differ between the two groups at the peak ajmaline dose (Table 2).

## 3. Discussion

### 3.1. Main Findings

In this study, we show that (1) the prevalence of a left axis deviation during ajmaline provocation testing in patients with a positive ajmaline test is 17.5% and (2) that this left axis deviation is not caused by conduction slowing in the RVOT but is due to additional conduction slowing in the more proximal conduction system. 

The observed left axis deviation could possibly occur due to excessive conduction slowing in the RVOT, as this is the most commonly affected region in BrS patients [1,4]. Our results, however, show that the RVOT conduction, as mirrored by the conduction slowing in the 4th quartile of the QRS-loop, slows in equal amounts between patients with a left axis deviation and patients without a left axis deviation while they develop the type-1 ECG. This demonstrates that the RVOT region is equally affected in both groups and that the cause of the left axis deviation must originate more proximally in the conduction system. Conduction slowing in BrS patients who developed a left axis deviation was indeed most prominent in the 1st and 3rd quartiles of the QRS-loop, most likely representing additional septal and free wall depolarization abnormalities, respectively [7,10,11,12]. Apparently, in these patients with a left axis deviation, conduction seems to be more extensively affected. This could potentially explain some part of the varying arrhythmogenic risk in BrS-patients [1]. These conductional differences cannot be explained from our results by the currently known (likely) pathogenic *SCN5A* mutation, as the mutation status did not differ significantly between groups. Still, loss-of-function *SCN5A* mutations will often result in a more general conduction disease. Surprisingly, at baseline, conduction in the 3rd quartile is slightly faster in patients with a left axis deviation, whilst out of the four quarters, this quartile showed the most conduction slowing at the peak ajmaline. If conduction in the free wall actually is the most extensively affected region, this faster conduction at baseline is remarkable and against our expectations. The underlying mechanism and the clinical implications of this finding are unclear and could be the focus of future research. In addition, global repolarization appeared to be more discordant from depolarization in patients with a left axis deviation, as indicated by increased, borderline abnormal (>105–135°), QRST-angles. In the general population, abnormal (>135°) and borderline abnormal (105–135°) QRS-T angles are strong predictors for cardiac death. One could therefore hypothesize that patients with a positive test with a concomitant left axis deviation might have a higher arrhythmogenic risk.

As to the origin of the characteristic ST-elevation (or J-point elevation) that is associated with the development of a type-1 BrS-ECG, previous studies suggested that excitation failure at the RVOT was elementary in this process [13,14]. In the current study, the amount of ST-elevation did not differ between those ajmaline-positive patients with or without a left axis deviation upon ajmaline provocation. Whether vectorcardiography, which is dependent on spatial vectors, would be able to add insights to the occurrence and localization of an excitation failure in the development of the type-1 ECG during the simultaneous slowing of conduction during type-1 ECG development is currently uncertain. 

### 3.2. Future Perspective

Future studies may investigate whether the occurrence of a left axis deviation during ajmaline testing in BrS-patients partly determines their arrhythmogenic risk. Clearly, a greater ability to stratify the arrhythmogenic risk in BrS patients would make it possible to optimize treatments accordingly.

### 3.3. Limitations

Vectorcardiograms were not recorded using additional orthogonal Frank XYZ leads but were reconstructed from 12-lead ECGs. Despite the fact that the reconstruction process using matrix multiplication has been validated, we cannot exclude some degree of variability from Frank leads [15]. However, we used the same lead setup in every patient and compared within-patient changes. In addition, by using the reconstructed vectorcardiograms, we enabled a future comparison of our data with other cohorts for whom digital 12-lead ECG data is available. 

In addition, as this was a retrospective exploratory study, we were not informed about the follow-up data of these patients so as to determine potential differences in arrhythmogenic risk or differences in the progression of the clinical phenotype as indicated by the presence or absence of a (ajmaline-induced) left axis deviation. 

## 4. Materials and Methods

### 4.1. Patients and Sodium Channel Provocation Testing

In this study, out of a cohort of 1430 patients who underwent provocation testing during the period from 2009 to 2015, a selection of patients was made based on the provocation test result and the cardiac axis. All patients for whom the provocation test was positive (see below) and whose electrocardiogram were available for a vectorcardiogram reconstruction were selected for this study; those with an abnormal or negative test were excluded. Tests were defined as (1) positive if a type-1 BrS-ECG occurred [1], (2) abnormal if arrhythmias or an excessive QRS-widening of ≥40% occurred, and (3) negative if the target dose was reached and none of the criteria above were met. Ajmaline was used as the sodium channel blocker and was infused intravenously in boluses of 10 mg/min until the maximum dose (1 mg/kg) was reached or until a positive or abnormal test result was obtained. None of the patients had exhibited a spontaneous type-1 BrS ECG before the provocation test. These patients underwent provocation testing because of symptoms (e.g., unexplained syncope or documented ventricular arrhythmias), a baseline ECG that raised the suspicion of BrS, family screening for BrS or family screening in the context of a sudden cardiac death or sudden unexplained death. 

### 4.2. Electrocardiographic Recordings, Analysis and Definitions

#### 4.2.1. Electrocardiographic Recordings

Modified 12-lead ECGs—with V3 and V5 placed cranially to V1 and V2 over the third intercostal space—were recorded at baseline and at one minute after each ajmaline bolus. 

#### 4.2.2. Electrocardiographic Analysis

ECGs were analyzed with the electrocardiographic analysis system MEANS, and its markers settings (e.g., P-wave onset, P-wave offset, QRS onset, etc.) were manually inspected and adjusted if deemed necessary [16]. Vectorcardiograms were reconstructed from the 12-lead ECG using the matrix multiplication as previously described [15]. The vectorcardiographic analysis consisted of dividing the spatial QRS-loop in four quartiles of equal length. Subsequently, the durations (ms) in the transverse plane were measured out of these four quartiles. Furthermore, to study the depolarization and repolarization interaction, the spatial QRS-T angle (°) and the vector magnitude (mV·ms) (i.e., ventricular gradient) of the spatial QRS-T integral were determined. An abnormally increased QRS-T angle indicates global discordant repolarization, values of 105–135° (borderline abnormal) and >135 (abnormal) are associated with fatal cardiac arrhythmias in the general population [8]. The ventricular gradient is considered a three-dimensional measure of the heterogeneity of the action potential morphology [9]. In order to calculate the change in the conduction intervals, the vectorcardiographic parameters at baseline were deducted from the parameters at the peak ajmaline dose. To further evaluate the four quartiles of the QRS-loop between patients with a left axis deviation and patients without a left axis deviation, the proportion of patients with an increase above the median value of the entire cohort was compared between the two groups.

#### 4.2.3. Electrocardiographic Definitions

A left axis deviation was defined in this study as a leftward shift of the frontal QRS axis of >30° at the peak ajmaline dose as compared to the baseline. We defined the fourth quartile of the QRS-loop—in patients with a positive ajmaline test—as representative of RVOT conduction [4]. 

### 4.3. Statistical Analysis

The statistical analysis was performed with SPSS Statistics (version 25.0, IBM Corporation, Armonk, New York, USA). Categorical variables are presented as frequencies and group percentages. To compare such variables, the Fisher-exact test was used. Continuous variables are expressed as the mean ± standard deviation in the case of a normal distribution or the median [interquartile range] in the case of a skewed distribution. Histograms and Q–Q plots were used to evaluate the distribution of variables with continuous data. The unpaired two-tailed t-test was used to compare normally distributed variables; in the case of a skewed distribution, the Mann–Whitney U test was used. A *p*-value of <0.05 was accepted as the level of statistical significance. 

## 5. Conclusions

In BrS-patients, a left axis deviation during ajmaline testing occurs in a significant number of patients. This leftward shift in axis is not caused by an additional conduction slowing of the RVOT but appears to occur as a consequence of an additional, more proximal conduction slowing. Whether a left axis deviation can also be used for the stratification of arrhythmia risk is currently undetermined. 

## Figures and Tables

**Table 1:** Baseline characteristics.

Characteristic	Left Axis Deviation (*n* = 56)	No Left Axis Deviation (*n* = 264)
Age, years	46 ± 13	45 ± 13
Male sex	20 (35.7) *	143 (54.2)
Length, cm	171 ± 10 *	175 ± 11
Weight, kg	71 ± 16 *	78 ± 14
BMI, kg/m^2^	24 ± 4	26 ± 4
History of SVT, *n* (%)	0 (0.0)	6 (2.6)
History of VT/VF, *n* (%)	1 (2.2)	11 (4.8)
History of Syncope, *n* (%)	13 (28.9)	45 (20.4)
Family history of SCD/ SUD, *n* (%)	25 (47.2)	134 (52.3)
Family history of BrS, *n* (%)	31 (55.4)	135 (51.1)
Genetic testing, *n* (%)	35 (62.5)	154 (58.3)
Likely pathogenic or pathogenic *SCN5A* variant, *n* (%)	9 (25.7 ^‡^)	18 (11.7 ^‡^)
Indication for Test		
ECG suspicious for BrS, *n* (%)	7 (12.5)	56 (21.2)
Symptoms (Syncope, VT/VF or AF), *n* (%)	6 (10.7)	18 (6.8)
Family screening BrS, *n* (%)	32 (57.1)	134 (50.8)
Family screening SCD/ SUD, *n* (%)	11 (19.6)	56 (21.2)
Ajmaline administered (mg)	72 ± 19	74 ± 24
Percentage of maximum ajmaline target dose administered (%)	100 ± 22	95 ± 28
ECG Parameters		
Heart rate (bpm)	66 ± 10	68 ± 1
PR-interval	168 ± 30	168 ± 29
QRS-duration (ms)	100 ± 14 *	105 ± 15
QTc-interval (ms)	425 ± 22	424 ± 28
Normal QRS-axis, *n* (%)	53 (94.6)	224 (84.8)
Left QRS-axis, *n* (%)	3 (5.4)	23 (8.7)

Data are presented as the mean±SD or *n* (%).* = *p* < 0.05. ^‡^: of patients who underwent genetic testing, nonsignificant (*p* = 0.057). BMI: body mass index, BrS: Brugada syndrome, SCD: sudden cardiac death, SVT: supraventricular tachycardia, SUD: sudden unexplained death, VT: ventricular tachycardia, VF: ventricular fibrillation.

**Table 2:** Vectorcardiographic parameters in patients with a left axis deviation of >30° at baseline, at ajmaline peak, and the change between the baseline and ajmaline peak.

Characteristic	Baseline		Peak		Change in Vectorcardiographic Parameters between the Baseline and Ajmaline Peak	
	Left Axis Deviation (*n* = 56)	No Left Axis Deviation (*n* = 264)	Left Axis Deviation (*n* = 56)	No Left Axis Deviation (*n* = 264)	Left Axis Deviation (*n* = 56)	No Left Axis Deviation (*n* = 264)
QRS duration, ms	100 ± 14 *	105 ± 15	143 ± 21 *	136 ± 20	+43 ± 16 ^†^	+31 ± 21
Duration of q1, ms	33 ± 4	33 ± 5	45 ± 8 *	42 ± 8	+12[8;18] ^†^	+8[4;12]
Duration of q2, ms	15 ± 3	15 ± 3	18 ± 6 *	20 ± 5	+2[–2;6] ^†^	+6[2;8]
Duration of q3, ms	10[8;12] *	12[10;14]	22[14;30] ^†^	14[10;20]	+12 ± 10 ^†^	+5 ± 7
Duration of q4, ms	42 ± 10	44 ± 11	58 ± 16	57 ± 14	+17 ± 14	+13 ± 15
Frontal axis QRS-loop, °	29 ± 24	29 ± 36	–74[–107;3] ^†^	29[0;40]	–98[–128;–24] ^†^	–6[–27;–1]
Spatial QRS T angle, °	67 ± 27	62 ± 34	122[59;147] ^†^	44[25;91]	39 ± 46 ^†^	–1 ± 43
Ventricular gradient, mV.ms	37 ± 16	41 ± 17	35 ± 13	38 ± 14	–1 ± 7	–3 ± 7
Max. J-amplitude in V1-V2ic3 (µV)	98.5[59;129]	89[52;142]	311[220;397]	342[245;440]	236 ± 185	246 ± 165

Data are presented as the mean±SD. median [IQR]. Max.: maximum; ms: milliseconds; mV: Millivolts; μV: Microvolts; q1: first quartile of the QRS-loop; q2: second quartile of the QRS-loop; q3: third quartile of the QRS-loop; q4: fourth quartile of the QRS-loop. * = *p <* 0.05 and ^†^ = *p* ≤ 0.001 for patients with a left axis deviation compared to patients with no left axis deviation.

## Data Availability

The data presented in this study are available on request from the corresponding author. The data are not publicly available due to privacy reasons.

## Abbreviations

BrS	Brugada Syndrome
ECG	Electrocardiogram
